# Managing the risk of Hendra virus spillover in Australia using ecological approaches: A report on three community juries

**DOI:** 10.1371/journal.pone.0209798

**Published:** 2018-12-31

**Authors:** Chris Degeling, Gwendolyn L. Gilbert, Edward Annand, Melanie Taylor, Michael G. Walsh, Michael P. Ward, Andrew Wilson, Jane Johnson

**Affiliations:** 1 Australian Centre for Health Engagement Evidence and Values, School of Health and Society, University of Wollongong, Wollongong, NSW, Australia; 2 Sydney Health Ethics, School of Public Health, University of Sydney, Sydney, NSW, Australia; 3 Marie Bashir Institute for Infectious Disease and Biosecurity, University of Sydney, Sydney, NSW, Australia; 4 Sydney School of Veterinary Science, University of Sydney, Camden, NSW, Australia; 5 EquiEpiVet, Picton, NSW, Australia; 6 Department of Psychology, Macquarie University, Sydney, NSW, Australia; 7 Westmead Clinical School, Sydney Medical School, University of Sydney, Westmead, NSW, Australia; 8 Menzies Centre for Health Policy, University of Sydney, Sydney, NSW, Australia; Kyoto University, JAPAN

## Abstract

**Background:**

Hendra virus (HeV) infection is endemic in Australian flying-fox populations. Habitat loss has increased the peri-urban presence of flying-foxes, increasing the risk of contact and therefore viral ‘spillovers’ into horse and human populations. An equine vaccine is available and horse-husbandry practices that minimize HeV exposure are encouraged, but their adoption is suboptimal. Ecological approaches–such as habitat creation and conservation–could complement vaccination and behavioural strategies by reducing spillover risks, but these are controversial.

**Methods:**

We convened three community juries (two regional; one metropolitan) to elicit the views of well-informed citizens on the acceptability of adding ecological approaches to current interventions for HeV risk. Thirty-one participants of diverse backgrounds, mixed genders and ages were recruited using random-digit-dialling. Each jury was presented with balanced factual evidence, given time to ask questions of expert presenters and, after deliberation, come to well-reasoned conclusions.

**Results:**

All juries voted unanimously that ecological strategies should be included in HeV risk management strategies but concluded that current interventions–including vaccination and changing horse-husbandry practices–must remain the priority. The key reasons given for adopting ecological approaches were: (i) they address underlying drivers of disease emergence, (ii) the potential to prevent spillover of other bat-borne pathogens, and (iii) there would be broader community benefits. Juries differed regarding the best mechanism to create/conserve flying-fox habitat: participants in regional centres favoured direct government action, whereas the metropolitan jury preferred to place the burden on landholders.

**Conclusions:**

Informed citizens acknowledge the value of addressing the drivers of bat-borne infectious risks but differ substantially as to the best implementation strategies. Ecological approaches to securing bat habitat could find broad social support in Australia, but disagreement about how best to achieve them indicates the need for negotiation with affected communities to co-develop fair, effective and locally appropriate policies.

## Introduction

Hendra virus (HeV) infection is endemic in Australian flying-fox populations. Of the four flying-fox species native to Australia, the spectacled flying-fox (*Pteropus conspicillatus*) and black flying-fox (*Pteropus alecto*) are considered the most likely sources of equine HeV infections [1, 2]. The current hypothesis is that the virus is transmitted to horses by mucous membrane exposure through the ingestion or inhalation of flying-fox excreta [most likely urine) from contaminated pasture or water [3, 4]. Horses can shed virus before developing clinical signs and the presentation of HeV disease is highly variable, non-specific and difficult to interpret [5, 6]. Horses also amplify the virus and can transmit HeV to other horses, dogs, and humans. While HeV infection appears to be relatively benign in domestic dogs, it causes catastrophic neurological and respiratory disease in humans and horses [7, 8]. Since first characterised in 1994, there have been sporadic ‘spillover’ events in Queensland and northern New South Wales (NSW) during which more than 100 horses have died or been euthanized following diagnosis. The relative infectivity of known HeV strains is low–effective transmission requires close contact with the blood and/or bodily fluids of infected horses and many people have been exposed to HeV-infected horses without zoonotic transmission. Conversely HeV is highly virulent and the case fatality rate in humans is high–four of the seven cases have died [4]. Horses may shed virus up to three days before developing clinical signs and early clinical signs in the horse are considered non-specific. No therapeutics have been registered for human use but experimental monoclonal antibodies have been used as a compassionate treatment for individuals with high HeV risk exposures [9, 10]. Not including the expense of managing human HeV cases and exposures, the cost and economic loss associated with each localised HeV outbreak has been estimated to be AUD $30,600 for each horse death [11].

Public health responses to HeV have focussed on education and behaviour modification amongst high-risk groups such as veterinarians, horse owners and people who work in equine industries [12]. Horse-husbandry practices that minimize the risk of HeV exposure–such as covering water troughs and fencing-off flowering and fruiting trees–have been widely publicised in the media and encouraged through targeted health and occupational safety messaging [13, 14]. New occupational safety regulations were introduced, requiring veterinarians attending sick horses to wear personal protective equipment and restrict clinical care to minimally invasive emergency care until the animal has been tested negative of HeV infection [15]. This process can take 12–64 hours, depending on the day of the week and location [16]. Because of the human deaths–and after the unprecedented number of equine cases in 2011 –development of an HeV vaccine for horses was prioritised, became commercially available on a minor use permit in 2012, and gained full registration in 2015 [17]. Vaccination has been advocated and defended as the most effective way of reducing the risk of HeV in horses—a Queensland Parliamentary Inquiry in 2016 confirmed the vaccine’s safety and efficacy. It is estimated that approximately 14% of the total Australian horse population has been vaccinated with 500,000 doses administered to close to 150,000 horses. However vaccine uptake remains suboptimal—even in areas deemed to be at higher risk of HeV spillovers [18]. Factors driving resistance to use of the vaccine include horse owners’ concerns about vaccine cost, safety and its potential impact on equine performance [19–21]. The adoption of safer horse-husbandry practices has also been less than ideal. Even though most owners appreciate the value of minimising the exposure of their horses to flying-foxes, many find the recommended changes to practices difficult to implement because of issues of practicality, the expense of introducing or changing infrastructure on their property, or both [14, 18, 22]. As horse-owner and community responses to the risks posed by HeV are variable–and the uptake of effective preventive interventions has been suboptimal–some veterinarians are refusing to attend unvaccinated horses, or are abandoning equine practice altogether because of overriding concerns for their personal safety and legal liability during HeV spillover events [23].

Climate change, habitat loss and habitat fragmentation are key stressors that drive emergence of new infectious diseases from wild animal populations [24, 25]. Queensland alone has lost 9.7 million hectares of forest through land clearing since the 1970s, with native vegetation cover reduced by at least 50% over the last 200 years [26]. Loss of habitat and depletion of native food sources contribute to flying-foxes moving into agricultural and peri-urban areas, increasing the risk of viral spillovers into horse and human populations [27, 28]. Nutritional stress may also have impacts on flying-fox physiology and their immune responses in ways that facilitate viral shedding [29–31]. Flying-foxes are highly mobile animals that may travel hundreds of kilometres to feed on their preferred food–the nectar of flowering eucalyptus trees [27]. As large areas in north-eastern Australia once covered by eucalypt forests have been cleared for agriculture [32, 33], flying-foxes are encroaching upon human settlements in search of anthropogenic food sources such as fruit trees. The impacts of climate change on flying-fox behaviours and distribution are predicted to markedly expand the geographic range of HeV risk [34]. It is increasingly clear that flying-fox ecology drives the epidemiological dynamics of HeV in bat populations and risk of spillover to horses and, consequently, humans [27, 31]. Increased contact between flying-foxes, domestic animals and human populations in these altered landscapes and disturbed ecosystems exposes the latter to novel pathogens such as HeV, creating the current public health challenge [28].

Ecological approaches could usefully complement HeV vaccination and changing horse husbandry practices, such that flying-fox habitat conservation could be an effective public health intervention [27]. Spectacled and grey-headed flying-fox (*Pteropus poliocephalus*) species have long been a protected species under state or national legislation, although enforcement was not well resourced or prioritised until the emergence of HeV [13, 35]. Community-driven and unregulated culling and roost dispersal were the primary modes for managing conflict with flying-fox populations [36]. Despite the introduction and enforcement of protective legislation, current land management strategies for flying-foxes still focus on removing resources to deter the establishment of roosts near human settlements [37]. Experience has shown that relocating flying-fox camps is expensive and almost always fails in the longer term [11, 38]. Rather than solving the problem, dispersal efforts can also lead to the establishment of permanent secondary camps in unexpected and undesirable areas [39]. Roost dispersal could also increase the risk of HeV transmission within the flying-fox population [40, 41]. Yet in a recent Queensland-wide survey of community attitudes, some respondents argued for the creation of habitat as a food resource for flying-fox populations as a means to reduce conflict with humans [42]. Land or habitat sparing and wildlife-friendly farming have been proposed as means to achieve ecological benefits from agricultural landscapes [43]. Habitat creation and conservation could reduce stress on flying-fox populations, and thereby the risks of zoonotic spillover events [40, 44]. However, a large proportion of the Australian public considers flying-foxes to be pests [42] and historically the prioritisation of measures that protect them have been met with considerable political resistance, ignored by governments charged with maintaining them, or both [13, 36, 45]. The adoption of ecological approaches to HeV risks could be highly controversial if they are perceived to place limits on economic development and restrict the rights of landholders [46, 47].

We report on three community juries convened to consider dilemmas raised by increased contact between humans and flying-foxes and the emergence of HeV as a threat to human and equine health. Our aim was to elicit the views of well-informed members of the Australian public regarding the acceptability and perceived legitimacy of adding ecological approaches to current interventions that mitigate the risk of HeV spillover. Gaining some understanding of the public acceptability (or otherwise) of ecological approaches is important because the adoption of these types of interventions for HeV risks requires substantial reform of land use policy. Previous research shows that a lack of consistency and public trust in Australian government planning decision-making have led to anti-government attitudes among many regional landholders [48, 49]. Moreover, the social response to HeV emergence in Australia has been recruited to promote other policy agendas and political objectives–including protecting agricultural land and asserting the rights of individuals to remove flying-foxes from their property [13]. Globally and in the Australian context, land management policy is increasingly turning from public regulation to market-based interventions to manage conflicts between environmental conservation and rural livelihood needs [47, 48, 50]. Even though neither land acquisition nor market-based approaches to habitat creation are being actively considered by policymakers, to mitigate bat-borne zoonotic risks the results of this study can inform nascent discussions about the economic and political feasibility of employing landscape management as a zoonotic disease control and prevention strategy.

This study was approved by the Human Research Ethics Committee at The University of Sydney and supported by a project grant (APP1083079) from the Australian National Health and Medical Research Council. The funding organization had no role in the design, conduct, analyses or reporting of this study.

## Materials & methods

### Design and study setting

A community jury is a group of citizens brought together to receive detailed evidence about–and deliberate on–a specific issue [51]. Unlike surveys and focus groups, the process involves extensive provision of information; constructive, structured dialogue between publics and experts; and adequate time for consideration [52]. The method assumes that people can think rationally and change their views should the evidence warrant it [53]. The process is like a legal proceeding, but the outputs are not legally binding: instead they provide evidence of public values and the likely acceptability and perceived legitimacy of different policy alternatives to assist policymaking [51, 52]. Community juries have been used in Australia and elsewhere to consider issues surrounding infectious disease control and prevention [54, 55], and changes in land use and environmental health risks [56, 57]. Community juries are a deliberative method, with these general characteristics:

1. A group of citizens is convened for 1–3 days;
2. They are asked to consider a specific issue;
3. They hear (sometimes opposing) evidence from experts, and are given an opportunity to ask them questions;
4. They are given time for deliberation, and to come to a conclusion, which is documented.

We convened three community juries: one in Rockhampton (Central Queensland), one in Lismore (Northern NSW) and one in Sydney (NSW). All were held over two days between October 2017 and March 2018. Rockhampton and Lismore are both small cities in rural areas on the eastern seaboard of Australia; both have been affected by local HeV outbreaks. The capital of NSW, Sydney is a coastal metropolis located further south than the other study settings. Home to a large racing and recreational horse population, Sydney has at least a dozen permanent flying-fox camps located across the metropolitan region [58]. Sydney is yet to be affected by an HeV outbreak but could be at increasing risk of a spillover event as the effects of climate change extend the range of the reservoir hosts further southward [34, 59].

### Participants and recruitment

We contracted an independent professional research service to recruit the jury participants, who lived in each of the study sites, from randomly generated list-based samples, based on representative socio-demographic characteristics (including gender and age) using random-digit-dialling. When first contacted by the recruitment company, potential participants were told that the study was about flying-foxes, horses and how best to manage infectious disease risks. The jurors were then selected purposively from the pool of people who indicated they were interested in taking part, with the final composition of each jury determined by individual availability and eligibility (satisfy exclusion and inclusion criteria). Each jury was stratified to ensure that people who owned or interacted with horses and people who had no interaction with horses were represented, and that there was socioeconomic and cultural diversity within each jury [60, 61]. Thirty-one people were recruited to act as jurors across the three study settings (Table 1).

**Table 1:** Characteristics of jury participants.

	Jury 1 Rockhampton (n = 12)	Jury 2 Lismore (n = 9)	Jury 3 Sydney (n = 10)
**Age (years)**			
18–34	5	1	3
35–54	4	4	4
> 55	3	4	3
**Gender**			
Male	6	5	3
Female	6	4	7
**Highest Educational Attainment**			
High School	4	2	2
Trade / Diploma	5	4	4
Bachelor Degree	2	2	3
Postgraduate Degree	1	0	1
**Horse Owner / Works with Horses**			
Owner	3	3	0
Not an owner	6	4	7
Not an owner but works with horses	3	2	3
Socio-Economic status of suburb*			
Low	2	9	2
Middle	10	0	4
High	0	0	4

* Based on Socio-economic Index for Area (SEIFA)

Clearly a jury of 10–14 people cannot possibly represent a statistically characterized sample of ‘the general public’. But it is possible to derive a sense of what an informed public would advise from a smaller group who are given factual information and time to deliberate [60, 62]. Rather than focus on ‘statistical representativeness’, participants are recruited to capture diversity of experiences and backgrounds in a community, and the deliberation processes organized so as to redress power imbalances as much as is feasible [63]. When conducted in this way, community juries can capture and reflect the key community concerns and arguments about current and proposed policy directions–i.e. what should be done to address a specific issue [64, 65]. Community characteristics were reflected in a more socially and culturally diverse jury in Sydney, than those in Rockhampton and Lismore, which are areas of lower socioeconomic advantage. All three juries included participants with different levels of educational attainment (Table 1). Jurors received a modest honorarium in recognition of their participation and contribution to jury processes and outcomes.

### The questions (‘charge’) put to the three community jurys

**Fig 1:**
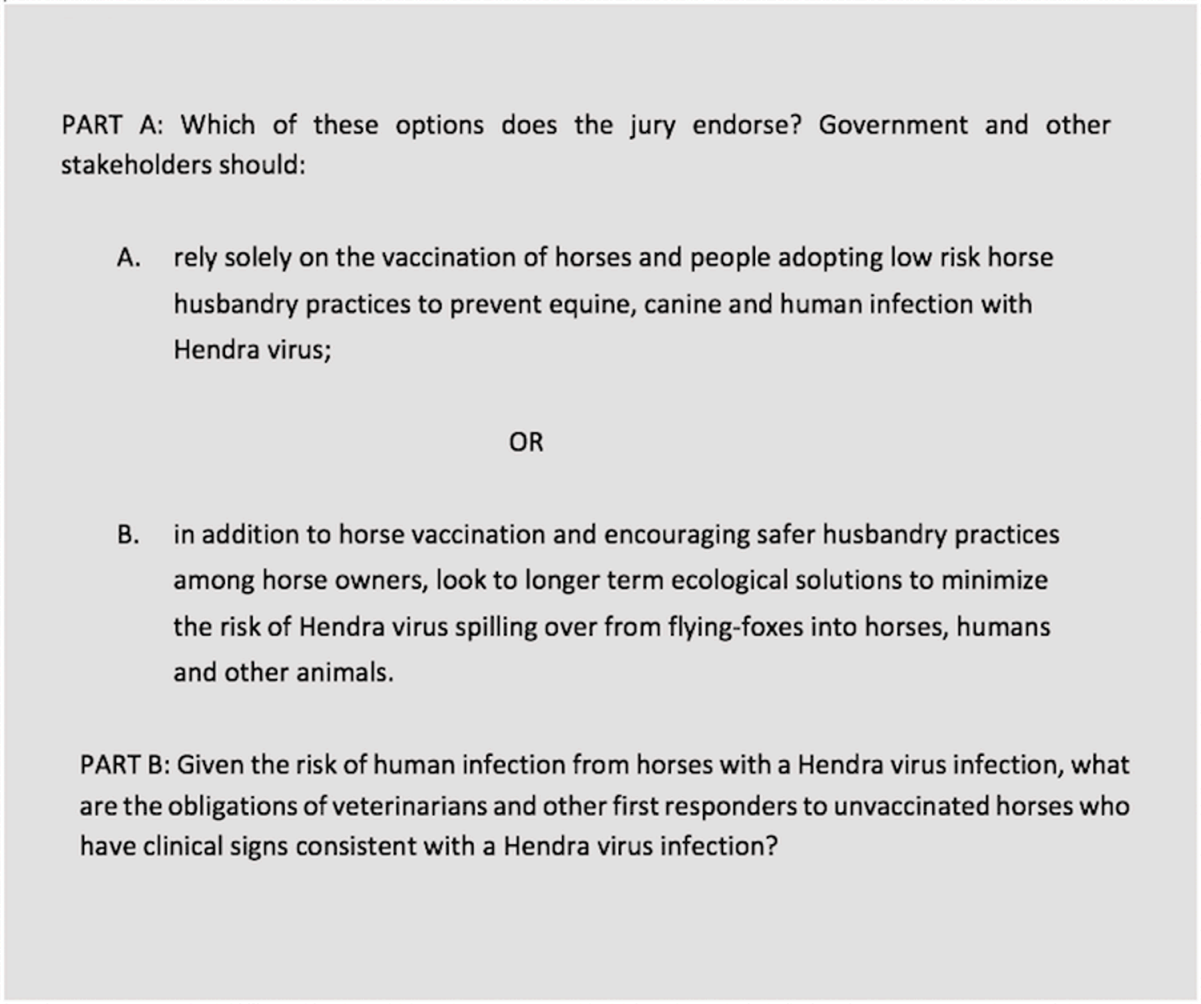
Questions for the community juries.

**Fig 2:**
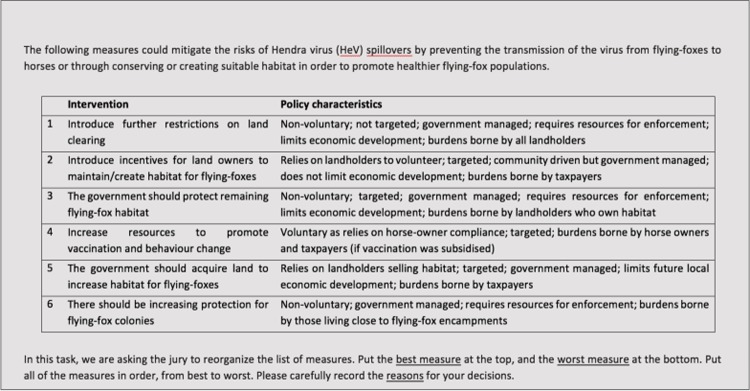
The prioritisation task for the community juries.

First, we asked each jury to consider the legitimacy and appropriateness of adding ecological approaches to current HeV control interventions (Fig 1). To validate the juries’ verdicts–and to provide some insight into the public acceptability of different mechanisms to promote ecological benefits–we then asked jurors to consider and prioritise six different potential interventions (Fig 2). Based on a review of the current policies and available peer-reviewed and grey literatures, the list included: measures directed towards mitigating proximal risks by committing more resources towards promoting the uptake of HeV vaccine or recommended horse husbandry practices by horse owners [17, 18, 66]; and measures focused on the distal drivers of disease emergence such as landscape management and flying-fox habitat creation and protection [27, 43, 44, 46, 67].

### Procedures

**Fig 3:**
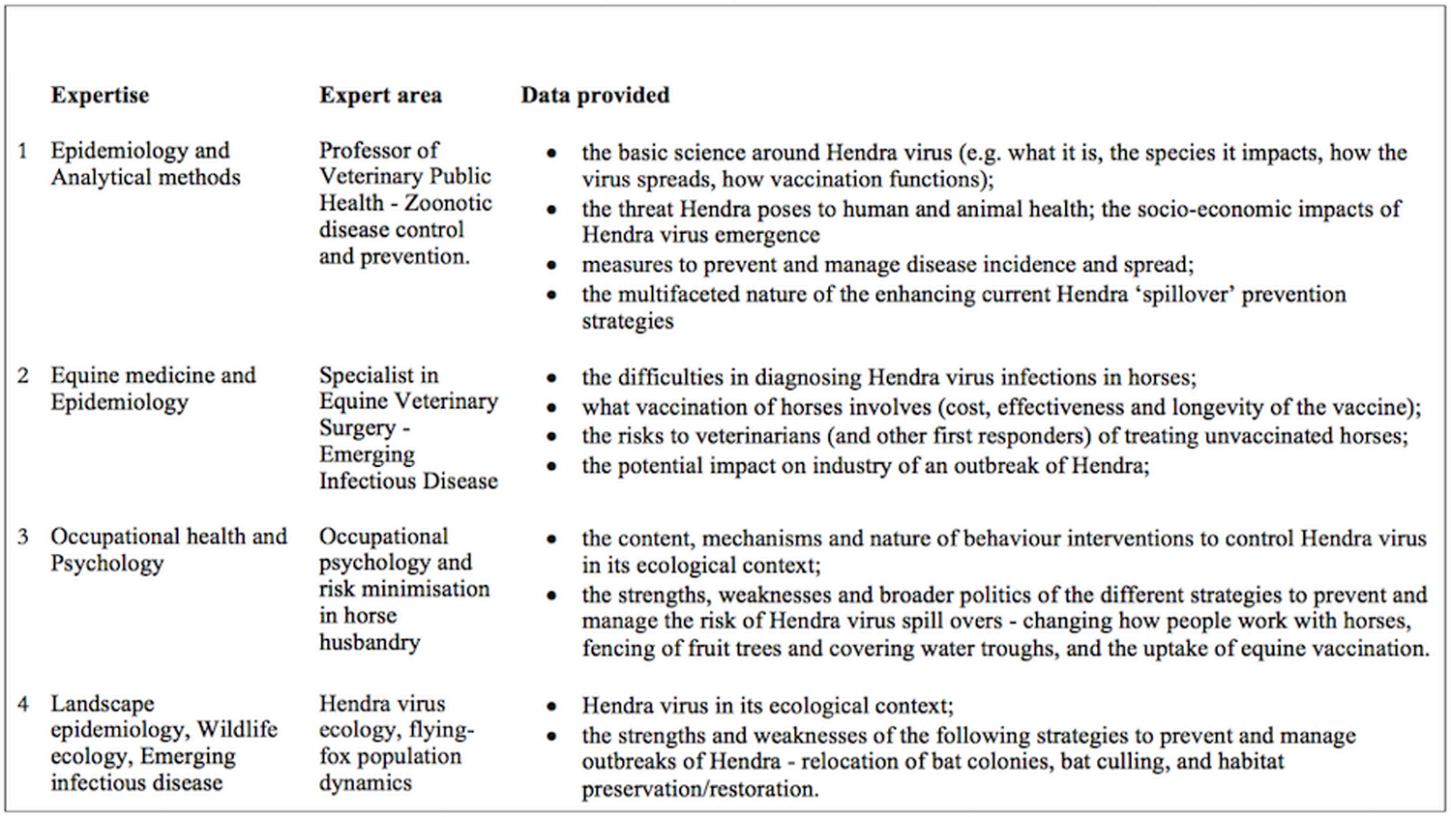
Expert testimony provided to the community juries.

Each jury commenced with an orientation session to introduce the process, the questions for consideration and obtain consent. Jury Day 1 focused on understanding: basic HeV biology; the socio-economic impact of HeV emergence; different interventions to manage infection risks; common clinical and practical issues associated with these strategies and with management of potentially HeV-infected horses; and the epidemiological evidence for the drivers of HeV emergence (Fig 3). Testimony from four experts was pre-recorded and shown to jurors as video presentations. Experts were selected on the basis of their institutional roles, experience and expertise, to provide balanced and factual information supporting different expert perspectives on the feasibility, benefits and potential cost of different strategies for managing HeV risk (equine vaccination; adopting low-risk horse husbandry practices; and ecological approaches). Each presentation ran for approximately 30 minutes. Pre-recording ensured the format of the evidence presented was standardized across juries. The experts’ bio-sketches and video presentations are available online [68]. After each video presentation the expert was available by teleconference call or in person for jurors to ask questions. These question and answer sessions, facilitated by a researcher, allowed jurors to clarify or question the evidence and opinions presented. Facilitation focused on promoting constructive dialogue and fair interaction amongst jurors.

For the first hour of Jury Day 2, jurors reflected on, discussed and debated the evidence, aided by a researcher acting as facilitator. Juries then deliberated for an hour, without researchers being present, to reach a verdict on the questions posed. The verdicts, underpinning reasoning and dissenting views, were reported to the research team in a final facilitated feedback session. Our research and reporting processes for these Community juries were cross-checked against the CJChecklist protocol [69].

### Data collection and analysis

The three deliberative groups (juries) are the units of analysis in this study. All jury deliberations (facilitated and un-facilitated) and expert question and answer sessions were audio-recorded and transcribed. To track changes in the positions held by individual jurors, participants completed an anonymous ballot at 3 time-points during jury proceedings (before they had been presented any evidence at the beginning of Day 1; after they had had time to consider this evidence at the end of Day 1; and after the deliberation and delivery of the verdict at the end of Day 2). Jurors also completed an Exit Survey for the purposes of process evaluation at the conclusion of each final jury session. During the final session a researcher recorded the verdict and reasons on a flipchart. Each point was reviewed by the jury to ensure accuracy. Transcripts were subsequently reviewed to identify and clarify key reasons why jurors supported or rejected the presented options. In what follows we have summarized jurors’ own descriptions of the rationale and reasoning that underpinned their responses to the questions asked of them. The verdict of the juries in response to the question (Fig 1 Part B) regarding what expectations the public should have of veterinary practitioners who are asked to respond to a potential HeV outbreak will be analysed separately and reported elsewhere.

## Results

**Fig 4:**
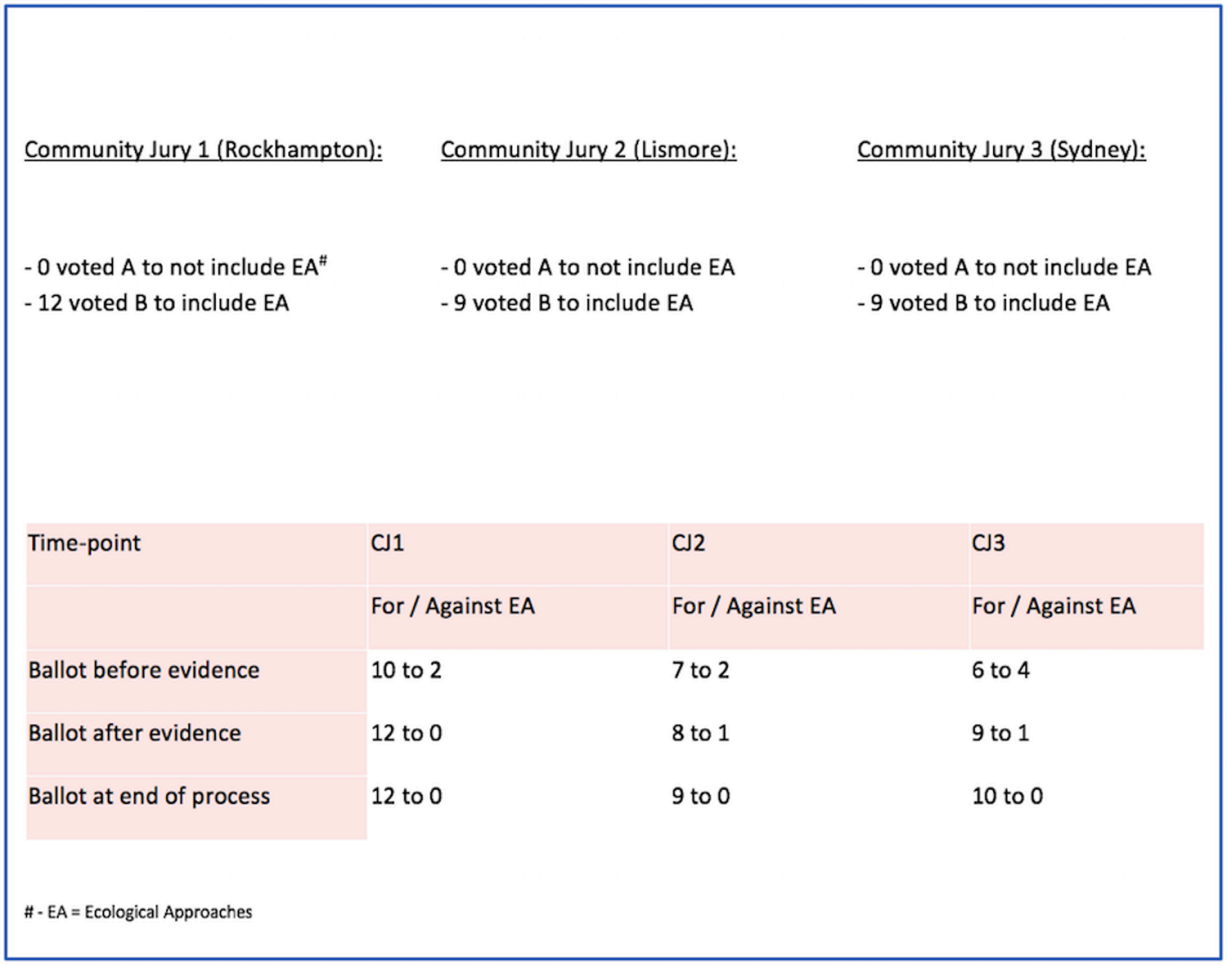
Final jury verdicts.

The juries in all three settings reported an unanimous verdict, namely that ecological strategies should be part of the approach taken to mitigate the risk of HeV spillovers in Australia. However, the balance of the vote changed during the course of each of the three juries before a final consensus was reached: at different time-points during proceedings some participants expressed the view that ecological approaches should not be actively pursued (Fig 4). The key reasons jurors gave for adopting ecological strategies as a complement to current approaches were: (i) because ecological approaches address underlying causes of disease emergence (rather than being a reactive response); (ii) because they might help to prevent the spillover of other as yet unknown flying-fox-borne pathogens; and, (iii) there would be broader benefits for the community. Jurors in all three study settings drew on widely held social norms such as the value of holistic approaches and upstream prevention to justify their decisions. About this, one juror in Lismore noted during deliberations:

Ecological changes have created this problem … So, you’ve got to go back to trying to remedy some of these ecological issues hand-in-hand with addressing the public health issues as well. This is going to be an ever-present risk. It’s not going to go away. (CJ2 - #9)

However, support for the adoption of ecological approaches was conditional. When asked to prioritise six different approaches to HeV risk mitigation, the further promotion of current measures such as equine vaccination and the adoption of safer horse-husbandry practices by owners were ranked as highest by all three juries (Table 2). Jurors held this position for reason of efficacy, efficiency and cost. Giving priority to measures that addressed immediate HeV risks was also seen as being an essential part of a process of community activation and awareness-raising in all three study settings. Jurors hoped that promoting vaccination and changing the horse-husbandry practices of owners would help the public understand the need for, and thereby be more willing to accept, the adoption of other ecologically-directed approaches to manage the risks of HeV spillover events. In reporting their verdicts all three juries emphasized the need for better communication about flying-fox behaviours, the ecological benefits they provide, and public education about the zoonotic risks they pose.

**Table 2:** The final rankings of each jury for different approaches.

Rank	CJ1- Rockhampton	CJ2—Lismore	CJ3 -Sydney
1	• Increase resources to promote vaccination and behaviour change	• Increase resources to promote [public awareness], vaccination and behaviour change (equal)	‧ Increase resources to promote [public awareness], vaccination and behaviour change AND • The government should protect remaining flying-fox habitat
2	• The government should acquire land to increase habitat for flying-foxes	‧ The government should protect remaining flying-fox habitat AND ‧ There should be increasing protection for flying-fox colonies AND • Introduce incentives for land owners to maintain / create habitat for flying-foxes	• Introduce further restrictions on land clearing
3	‧ The government should protect remaining flying-fox habitat AND • There should be increasing protection for flying-fox colonies	• Introduce further restrictions on land clearing	• Introduce incentives for land owners to maintain / create habitat for flying-foxes
4	• Introduce incentives for land owners to maintain & create habitat for flying-foxes	• The government should acquire land to increase habitat for flying-foxes	• There should be increasing protection for flying-fox colonies
5	• Introduce further restrictions on land clearing		• The government should acquire land to increase habitat for flying-foxes

So far there has not been a HeV spillover event in the Greater Sydney region. Notably, in their final rankings the jury in Sydney gave equal priority to measures that protect remaining flying-fox habitat and measures that promote vaccine uptake/risk reducing horse husbandry practices. They held that the adoption of at least one ecological measure should be prioritised because it would be a first step towards longer term measures to support ecological health. In contrast, in Rockhampton and Lismore the jurors’ endorsement of ecological approaches entirely depended upon sufficient resources being allocated to promote the vaccination of horses against HeV and the uptake of less risky husbandry practices among horse owners. They argued that equine vaccination provided effective and rapid protection against HeV transmission, and that measures to promote higher vaccination rates could be implemented immediately and were relatively cheap compared to financial and welfare costs associated with managing sick, unvaccinated, and potentially HeV infected horses. This final ranking was agreed to by all jury participants at both regional settings, noting that a minority of jurors at Lismore also believed that at least one of the ecological measures should be given the highest priority, but compromised during deliberations because the final outcome still distributed benefits and burdens between flying-fox populations, horse owners and landholders in a manner that they believed was appropriate.

Most jurors in all three study settings acknowledged that relying on owners to voluntarily vaccinate their horses was unlikely to be an effective stand-alone strategy. They noted that some owners were resistant to vaccination, others lacked the resources to prioritise this form of protection, and many would remain complacent unless a HeV spillover event occurred in their local area. A minority of participants on each of the three juries raised the possibility of making HeV vaccination mandatory, but this additional step was ultimately rejected by each of the deliberative groups for a range of reasons but primarily because of the economic and social costs of enforcement.

Different ecological approaches to mitigate the risks of HeV spillover events from flying-foxes were favoured in different settings (Table 2). The jury in Rockhampton preferred direct government responsibility and preventive actions. Jurors held that a publicly-funded, government run and managed program of land acquisition was the best mechanism to establish a network of green zones and increase habitat for flying-foxes because it would be most likely to produce the desired outcomes. Protecting flying-fox colonies and their habitats was ranked higher than the remaining strategies but it was not strongly endorsed by jurors, who noted that existing protections were fairly strict and that many people did not like living in and around colonies. As an overarching guiding principle, jurors held that farmers and other landholders should be allowed to manage their land to promote their own interests. Most also believed that the broader public, rather than the individual landholder, should bear the costs of conservation measures because the outcomes are a common good. Introducing incentives for land-owners to create flying-fox habitat was not strongly favoured because such a program would be expensive, difficult to manage and open to abuse and rorting. Finally, the jury in Rockhampton rejected the imposition of further restrictions on land clearing because it would be politically controversial and antagonise the rural community (and thereby derail attempts to implement other strategies that promoted flying-fox habitat creation and protection). During the final session of the jury in Rockhampton, a participant summed up the position taken by fellow jurors:

… there’s a lot of restrictions already. And if you’re going to put more restrictions on farmers they are going to be a lot less inclined to go along with anything. … And as we have all been saying, they are not following existing restrictions anyway (CJ1 - #7).

The Rockhampton jury saw an important role for government in providing funds to acquire flying-fox habitat. This jury was resistant to restrictive or coercive measures that impacted on individuals. In contrast, the Sydney jury preferred incentives and regulations to encourage individuals to take responsibility for avoiding exposure of themselves and others to HeV risks. A minority of jurors felt that additional measures would not be needed, if everyone vaccinated their horses and flying-fox habitat was protected by restricting land clearing. After considering other juror’s views, these participants eventually supported adoption of measures to generate ecological benefits, but only if they had broader environmental benefits than HeV risk mitigation alone; in common with the jurors in Rockhampton, they believed that flying-fox populations were already well protected and not necessarily endangered. Sydney jurors also rejected proposals for government to acquire land for flying-fox habitat; they felt that private land-holders should be expected to manage their land in ecologically sustainable ways, broadly defined. About this, a juror in Sydney noted:

… this seems to be a problem of getting landowners or land users to do the right thing. … Why should my tax money go into acquiring land for flying-fox habitat—the building the fences around trees, increasing restrictions on clearing so you're not taking away existing habitats, those measures I think are sufficient. (CJ3 - #10)

The Lismore jury ranked provision of more resources for vaccination and safer horse husbandry practices more highly than their Rockhampton and Sydney counterparts. However, a minority thought that ecological measures should be given equal priority because current approaches did not address the cause of the problem. They suggested combining increased protection of flying-foxes and their habitat (seen as equivalent) with incentives for landholders to create and maintain habitat on their properties, to stop deterioration in the short term and help reverse it in future. Like the Rockhampton jurors, most of those in Lismore felt it important that measures to encourage farmers to introduce mitigation strategies should be voluntary rather coercive. Notably, further restriction on private land clearing engendered heated debate and polarisation among jurors, in this setting: while some thought it vitally important, the majority felt there was already sufficient regulation and any escalation would require substantial buy-in from farmers and lead to controversy and further politicisation of the issue. Two of six jurors in Lismore who voted this way argued that further restrictions to land clearing should not be implemented under any circumstances. Of the remaining options, the least preferred was for the government to additional acquire land. The main objection was that governments already had substantial land holdings and the emphasis should be on identifying those considered to be suitable flying-fox habitat and improving their management, rather than adding more.

## Discussion

Recent research on the influence of flying-fox ecology and population dynamics on HeV spillovers suggests the possibility of landscape management as an effective public health intervention against this zoonotic risk [27, 28, 30]. The unanimous verdicts of our community juries suggest that ecological approaches to HeV risks are likely to be acceptable to citizens if they are informed about the costs, benefits and limitations of different types of intervention. Yet the acceptance of, and adherence by the population to, public health measures directed against infectious disease risks depend largely on the way people perceive the threat [70, 71]. When the juries’ verdicts are compared, those in Rockhampton and Lismore [which had been previously impacted by HeV spillovers) prioritised the need for reliable and rapidly effective prevention. The effect of proximity of HeV risk–on how each group prioritised different interventions–is consistent with previous research. Risk perceptions and previous personal experience are central to people’s decisions about how they protect themselves from infectious disease risks [72–75]. The conceptual foundations and goals of One Health had great appeal to jurors in all three settings and none of the jurors in Rockhampton or Lismore juries was indifferent to the ecological drivers of spillovers. However, the responses of both juries in these areas–where public health benefit from ecological approaches would be greatest–was clear. Ecological approaches to mitigate spillovers would be a luxury unless resources were also made available to mitigate proximal risks, such as by providing subsidised equine vaccination. Safeguarding human health must take priority over measures aimed at improving the health of flying-fox populations.

Jurors were surprised at the moderate uptake of HeV vaccination among horse owners. Research with horse owners suggests that drivers of HeV vaccine refusal are similar to those of vaccine refusal and hesitancy among parents. These include: distrust of scientific authority; different conceptualisations of how to act responsibly; and prioritisation of other values in decision-making around these issues [14, 22]. Public misunderstanding of risk is demonstrably important to vaccine refusal, and past communications about zoonotic risks from Australian flying-foxes have been variably effective [42, 76, 77]. However, experience of managing parental vaccine hesitancy and refusal suggests that the impact of “more scientific evidence” and “better public education” is limited [78, 79]. Many horse owners make their own judgements about the risks posed by HeV based on their proximity to flying-fox colonies and knowledge of previous spillover events in their area; and they may rely on measures other than vaccination to protect their animals from infection [19, 20, 22]. It was interesting that when told of limited equine immunisation rates, a minority of jurors (three or four) at each study site felt that vaccination was likely to be the most effective intervention, and, as such, making equine HeV vaccination compulsory would be appropriate. Disagreement among the jurors about the pragmatic value and ethics of compulsory HeV vaccination mirrored controversies and wider public health debates about how best to address falling vaccination rates in key human populations [80, 81]. Against a background of suboptimal vaccination uptake in high-risk areas, our results suggest that ongoing tensions between veterinarians and some horse owners on how to manage perceived HeV risks for horses could frustrate attempts to introduce ecological approaches. They also suggest that the politics of risk, and broader societal debates about individual freedom to abstain, conscientiously, from preventive health programs could complicate proposals for ecological approaches to HeV risk.

The different responses of each deliberative group to the prioritisation task were highly informative. Successful implementation of ecological approaches to HeV would also depend on navigating longstanding tensions in Australia about how best to manage and distribute the benefits and burdens of land use between privately-owned primary industries and taxpayers [82, 83]. In Rockhampton, an area where regulatory restrictions on land clearing have historically had significant electoral implications [32], the jury wanted the costs and burdens of any ecological approaches to be borne by government, because the outcome would be for broader public benefit. On the other hand, in metropolitan Sydney, the jury held that landholders should bear the cost of the upkeep of their assets and habitat protection and rehabilitation. The Lismore jury was divided on this question, eventually reaching a compromise that placed burdens on both landholders and governments. As these verdicts suggest, the politics of knowledge and different conceptions of responsibility for maintaining common goods are a constant component of these disputes [49]. Communities in regional and rural northern Australia can be particularly sensitive to centralised decision-making which is sometimes viewed as an incursion on the rights and interests of local residents [84–86]. People living on the land perceive that the academics and ‘city-dwellers’, proposing conservation measures, are ignorant of local conditions and the practical and moral implications of expecting landholders to solve environmental problems [47]. Increasing global competition and the struggle to maintain farm viability constrain landholders’ capacity to pursue positive environmental outcomes [87]. A common response to these arguments from policy-makers has been to argue for inclusion of local, experiential knowledge in decision-making about environmental management, but the jury outcomes suggest this may not be a panacea. The jury in Lismore was polarised around the appropriateness of placing further restrictions on land clearing. Notably, environmental concerns have become increasingly prominent in land management discourse in this region of NSW, gradually creating a constituency around the need to conserve natural habitats, and eroding the political power of farmers and rural landholders [88]. The move to empower local voices may not overcome heterogeneity and conflict within communities about how to enact effective environmental governance, but may actually reinforce existing divisions within the community [49, 89].

Our study has significant implications for those advocating landscape management to mitigate the risks of HeV spillover. Informed citizens in areas affected by HeV risks support the current emphasis on raising rates of equine HeV vaccination [17, 21]. Moves towards adoption of ecological approaches could find broad social support, but public polarisation around mechanisms for policy implementation points to the need to work with affected communities to co-develop fair, effective and locally appropriate solutions. Different interventions could lead to different distributions of the costs and burdens of prevention. As the Rockhampton jury identified, direct government action to achieve ecological benefits could effectively side-step the need to negotiate between opposing ideologies or embedded interests. Yet, experts claim that Australian governments do not have the capacity to maintain the continent’s ecological systems: for example, the combined network of government-managed protected habitat (National Parks and State reserves) is not sufficient to provide a secure future for nature over the longer term [90, 91]. Therefore, the greatest return for any conservation intervention would be on privately owned or leased lands. To be effective, ecological approaches need to be founded on collective action that involves both governments and landholders. Because of the impacts of local differences and political conflicts, previous engagement with landholders around how to manage their properties indicates they respond best to a mix of policy instruments [47, 92]. It is important, for the acceptability and perceived legitimacy of proposed methods for creating and conserving flying-fox habitat, that protection of the ecological value of the land is the aspiration of most, if not all, land managers in Australia; their attitudes tend to be anti-government rather than anti-environment [48, 85]. If ecological approaches are to be implemented, then governments and experts need to develop interventions that can be adapted to each landholder’s ecological and economic circumstances and to negotiate substantive and acceptable ways to support their underlying stewardship ethic [93].

### Strengths and limitations

Community juries are a deliberative method that involves a process of iterative two-way exchange of information between members of the public and experts. By providing extensive information from a range of experts, and ensuring conditions for reasonable and extended debate, community juries elicit more considered judgements than other social research methods such as surveys or focus groups. A possible limitation is that the sample size was small, but this is an unavoidable characteristic of community jury methods and is necessary for high-quality deliberation. It is also possible that each jury was comprised of participants who are more interested in issues surrounding flying-fox management than most Australia citizens, by self-selecting during otherwise random recruitment processes. The number of jurors in urban Sydney who own or regularly interact with horses was proportionally smaller than among regional juries, but was consistent with the socio-demographic characteristics of their respective populations. A strength of this study was the quality and reputation of the experts who gave testimony, and the process by which they moderated one another’s presentations until all experts could accept that all views presented could be argued from the evidence.

## Conclusions

Habitat sparing and wildlife-friendly farming have been proposed as measures that could promote ecological benefits for agricultural landscapes [43]. Researchers working on HeV ecology are beginning to explore the possibility of creating and conserving flying-fox habitat as a public health intervention against current and future zoonotic risks [27, 44]. Yet policy discussions about how best to manage the threats posed by zoonotic disease tend to focus on managing downstream risks and impacts [94, 95]. The failure of veterinary and human public health authorities, and wider political systems, to substantively engage with ecological approaches to zoonotic risks is understandable given how recently evidence for their potential efficacy is emerging [27, 28, 30]. Furthermore, there are challenges in understanding and modifying the complicated social and political structures that drive disease emergence [96, 97]. Our study shows that, apart from additional knowledge and evidence required for successful adoption of ecological approaches to HeV, they would require significant buy-in from multiple and diverse sets of stakeholders [27, 30]. The conceptual foundations and goals of One Health appealed to all three juries, but there were fundamental differences between them, in framing who is responsible for enacting these goals. This suggests that any attempt to use landscape management as a public health intervention will need to engage with, and negotiate, longstanding debates about the goals of land management in Australia. Recognition that regional populations are not uniform, and that community is as much a site of competition as cooperation, indicates a need to account for the particularities of place in land management and public health strategies. Because the effectiveness of ecological approaches to HeV spillovers depends on collective action towards flying-fox habitat creation and conservation across a large geographic area, local differences in HeV risk perception and attitudes to government action would need to be accommodated within the design of the intervention.
